# Young Parents

**Published:** 1889-08

**Authors:** E. McD. Bridgeford

**Affiliations:** 1914 N. 11th Street; St. Louis, Mo.


### CORRESPONDENCE.

### YOUNG PARENTS.

St. Louis, Mo., August 1, 1889.

*Ed. Daniel's Texas Medical Journal:*

In response to the inquiry of Dr. Samuel Fox, of Plantersville, Texas, I will say I do not know of a younger mother, than the case reported by him, but I recall a case, that came under my observation before I commenced the study of medicine, in which the mother was hardly thirteen years old, and the father was a little less than fifteen. The child was born at full term and lived about four months. Yours, Truly,

E. McD. Bridgeford, Mo.,

1914 N. 11th Street.

